# Supplementary material for: Multi-pathway Protective Effects of MicroRNAs on Human Chondrocytes in an In Vitro Model of Osteoarthritis
Source: Mol Ther Nucleic Acids. 2019 Jul 26;17:776–90. doi: 10.1016/j.omtn.2019.07.011 (PMC6716067; doi:10.1016/j.omtn.2019.07.011)
Supplement: Document S1. Tables S1 and S2 [file mmc1.pdf]

OMTN, Volume *17*

Supplemental Information

Multi-pathway Protective Effects of MicroRNAs on Human Chondrocytes in an *In Vitro* Model of Osteoarthritis

Rua Nader Al-Modawi, Jan E. Brinchmann, and Tommy A. Karlsen

Supplementary 1: Shared proteins between the three miRNAs

Common downregulated between miR-140-5p and miR140-3p	Common upregulated between miR-140-5p and miR140-3p	Common downregulated between miR-140-5p and miR146a	Common upregulated between miR-140-3p and miR146a
STA5A	STAT3	HCFC1	PCOC1
C1R	MEP50		
	NUP93		

Supplementary 2: Taqman probes, miRNA mimics and antibodies

Taqman Assays

Official symbol	Official full name	Company	Cat no.
IL6	Interleukin-6	Thermo Fisher Scientific	Hs00985639_m1
IL8	Interleukin-8	Thermo Fisher Scientific	Hs00174103_m1
IL1β	Interleukin 1-beta	Thermo Fisher Scientific	Hs01555410_m1
MMP13	Matrix metallopeptidase 13	Thermo Fisher	Hs00233992_m1
ADAMTS5	A disintigrin and metallopeptidase with thrombospondin type 1 motif, 5	Thermo Fisher Scientific	Hs00199841_m1
GAPDH	Glyceraldehyde-3-phosphate dehydrogenase	Thermo Fisher Scientific	Hs99999905_m1
miR-140-5p	microRNA 140-5p	Thermo Fisher Scientific	001187
U18	U18 small nuclear RNA	Thermo Fisher Scientific	001204
miR140-3p	microRNA 140-3p	Thermo Fisher Scientific	002234
miR146a	microRNA 146a	Thermo Fisher Scientific	00468
GABARAP	Gamma-aminobutyric acid receptor-associated protein	Thermo Fisher Scientific	Hs01572686_m1
CIR	Complement C1r subcomponent	Thermo Fisher Scientific	Hs00354278_m1
STAT5	Signal transducer and activator of transcription 5A	Thermo Fisher Scientific	Hs00559637_g1
RALA	Ras-related protein Ral-A	Thermo Fisher	Hs00800233_s1

		Scientific	
DHCR24	Delta(24)-sterol reductase	Thermo Fisher Scientific	Hs00207388_m1
LAMTOR5	Ragulator complex protein LAMTOR5	Thermo Fisher Scientific	Hs00246261_m1
BROX	BRO1 domain-containing protein	Thermo Fisher Scientific	Hs01013625_m1
DDAH1	N(G),N(G)-dimethylarginine dimethylaminohydrolase 1	Thermo Fisher Scientific	Hs00201707_m1
RIPK2	Receptor-interacting serine/threonine-protein kinase 2	Thermo Fisher Scientific	Hs01572686_m1
STAT2	Signal transducer and activator of transcription 2	Thermo Fisher Scientific	Hs01013115_g1
TP5313	Quinone oxidoreductase PIG3	Thermo Fisher Scientific	Hs00936519_m1

microRNA mimics

**Name**		**Company**	**Cat no.**
Pre-negative control no. 1		Thermo Fisher Scientific	AM17110
Pre-miR-140-5p	microRNA-140-5p	Thermo Fisher Scientific	PM10205
Pre-miR-140-3p	microRNA-140-3p	Thermo Fisher Scientific	PM12503
Pre-miR-146a	microRNA-146a	Thermo Fisher Scientific	PM10722

Antibodies (western blot)

**Primary antibodies**	**Concentration**	**Company**	**Cat.no**
Mouse anti-ACTB	1:1000	Abcam	ab8226
Rabbit anti-IL8	1:1000	Millipore	AB1427
Rabbit anti-IL6	1:1000	Abcam	Ab32530
Mouse anti-TRAF6	1:1000	Abcam	Ab40675
Rabbit anti-RALA	1:2000	Abcam	Ab126627
Rabbit anti-GBRAP	1:1000	Cell signaling	# 13733
Rabbit anti-RIPK2	1:1000	Abcam	Ab8428

Rabbit anti-NFκB-P65	1:1600	Abcam	Ab16502
Rabbit anti-LAMTORC	1:1000	Cell signaling	#14633
Rabbit anti-PDCD4	1:10000	Abcam	Ab80590
**Secondary antibodies**			
Goat anti-Rabbit (HRP)	1:5000<br>1:2000(when used with primary rabbit anti-PDCD4)	Vector labs	PI-1000
Horse anti-Mouse (HRP)	1:2000	Vector labs	PI-2000
