# Supplementary material for: Multi-pathway Protective Effects of MicroRNAs on Human Chondrocytes in an In Vitro Model of Osteoarthritis
Source: Mol Ther Nucleic Acids. 2019 Jul 26;17:776–90. doi: 10.1016/j.omtn.2019.07.011 (PMC6716067; doi:10.1016/j.omtn.2019.07.011)
Supplement: Document S2. Article plus Supplemental Infomration [file mmc2.pdf]

Molecular Therapy
Nucleic Acids
Original Article

Multi-pathway Protective Effects of MicroRNAs on Human Chondrocytes in an *In Vitro* Model of Osteoarthritis

Rua Nader Al-Modawi,[1] Jan E. Brinchmann,[1,2] and Tommy A. Karlsen[1]

[1]Norwegian Center for Stem Cell Research, Department of Immunology and Transfusion Medicine, Oslo University Hospital Rikshospitalet, Oslo, Norway; [2]Department of Molecular Medicine, University of Oslo, Oslo, Norway

Osteoarthritis (OA) is the most common degenerative joint disease. One of the main pathogenic factors of OA is thought to be inflammation. Other factors associated with OA are dysregulation of microRNAs, reduced autophagic activity, oxidative stress, and altered metabolism. microRNAs are small non-coding RNAs that are powerful regulators of gene expression. miR-140-5p is considered a cartilage-specific microRNA, is necessary for *in vitro* chondrogenesis, has anti-inflammatory properties, and is downregulated in osteoarthritic cartilage. Its passenger strand, miR-140-3p, is the most highly expressed microRNA in healthy cartilage and increases during *in vitro* chondrogenesis. miR-146a is a well-known anti-inflammatory microRNA. Several studies have illustrated its role in OA and autoimmune diseases. We show that, when human chondrocytes were transfected individually with miR-140-5p, miR-140-3p, or miR-146a prior to stimulation with interleukin-1 beta and tumor factor necrosis-alpha as an inflammatory model of OA, each of these microRNAs exhibited similar protective effects. Mass spectrometry analysis provided an insight to the altered proteome. All three microRNAs downregulated important inflammatory mediators. In addition, they affected different proteins belonging to the same biological processes, suggesting an overall inhibition of inflammation and oxidative stress, enhancement of autophagy, and restoration of other homeostatic cellular mechanisms, including metabolism.

INTRODUCTION

Osteoarthritis (OA) is the most common degenerative joint disease, affecting 10%–13% of adults in western countries.[1,2] There is yet no disease modifying treatment available. Patients with OA suffer pain, limited mobility, and reduced quality of life and often end up having joint replacement surgery. The exact causes of OA are unknown, but several risk factors have been identified, such as age, trauma, obesity, genetics, and other joint pathologies.[3] Inflammation is considered a major factor associated with the risk of cartilage loss and OA perpetuation.[4,5] At the molecular level, cartilage destruction occurs through the combined activities of cartilage degradation enzymes and inflammatory mediators. Increased levels of the inflammatory cytokines interleukin 1 beta (IL-1β) and tumor necrosis factor alpha (TNF-α) in the joint fluid have therefore been associated with the development of OA.[4,6,7] Autophagy is an essential mechanism that ensures cellular homeostasis by degrading and recycling cellular components. Autophagy regulates expression of inflammatory cytokines, is compromised in aging cartilage,[8,9] is defective in human OA chondrocytes and animal OA models, and can be regulated by microRNAs (miRNAs).[10–13]

miRNAs are small double-stranded non-coding RNAs that regulate gene expression in a sequence-based manner.[14] The 5′ strand is known as the leading strand and the 3′ strand is called the passenger strand. Usually the leading strand is the functional strand, but sometimes both strands can regulate gene expression.[15] Emerging evidence shows that one miRNA can target up to 100 genes, and one gene can be regulated by several miRNAs.[16,17] miRNAs are thus potent post-transcriptional regulators of gene expression and are implicated in several human diseases, including OA and other arthritic diseases.[18–20] miRNAs are therefore highly relevant as therapeutic molecules. miR-140 has been considered a cartilage-specific miRNA because it is predominantly expressed in cartilaginous tissue during development.[21] Knockout studies showed miR-140 to be protective against OA development.[22] There is ample evidence to suggest that both the 5′ strand (miR-140-5p) and the 3′-strand (miR-140-3p) are important for chondrogenesis and the biogenesis of OA. Both strands are highly expressed in healthy cartilage, miR-140-3p higher than miR-140-5p,[23] and downregulated in OA cartilage and synovial fluid.[24,25] Both strands are highly upregulated during *in vitro* chondrogenesis.[26,27] Previously, we showed that miR-140-5p was essential for SOX9 expression, and thus for chondrogenesis, and identified RALA as a direct target.[26] Additionally, we demonstrated that miR-140-5p has anti-inflammatory properties by targeting several proteins in the nuclear factor κB

Received 29 December 2018; accepted 16 July 2019;
https://doi.org/10.1016/j.omtn.2019.07.011.

Correspondence: Rua Nader Al-Modawi, MSc, Norwegian Center for Stem Cell Research, Department of Immunology and Transfusion Medicine, Oslo University Hospital Rikshospitalet, P.O. Box 4950 Nydalen, 0424 Oslo, Norway.
E-mail: runaal@rr-research.no
Correspondence: Jan E. Brinchmann, MD, PhD, Norwegian Center for Stem Cell Research, Department of Immunology and Transfusion Medicine, Oslo University Hospital Rikshospitalet, P.O. Box 4950 Nydalen, 0424 Oslo, Norway.
E-mail: jan.brinchmann@rr-research.no

 © 2019 The Authors.
This is an open access article under the CC BY license (http://creativecommons.org/licenses/by/4.0/).

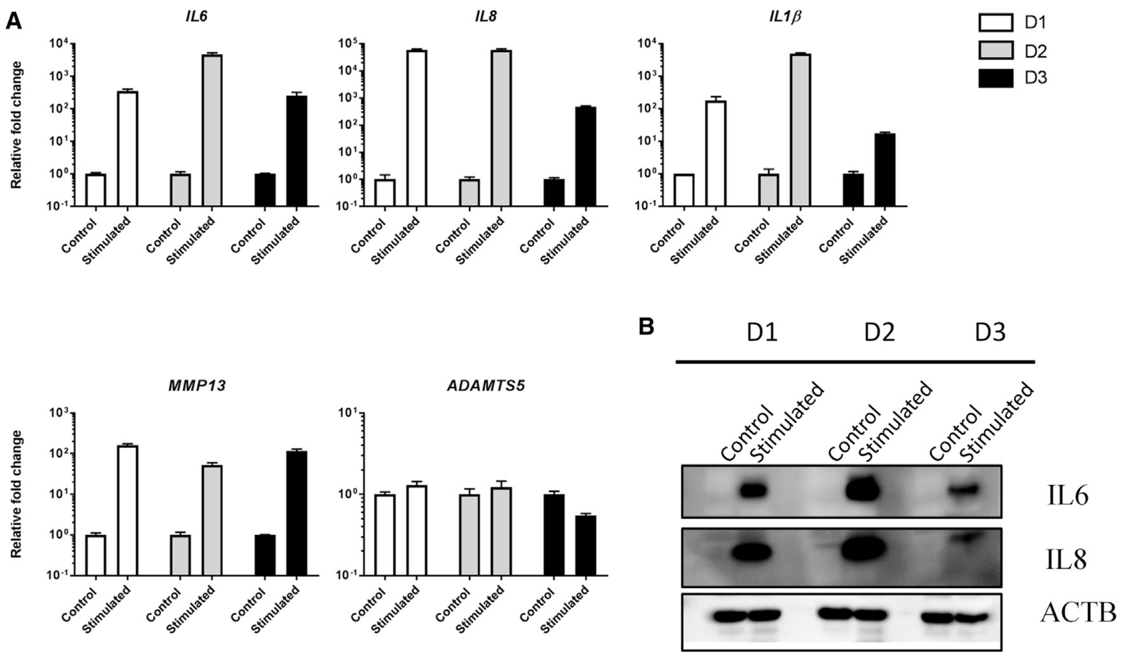

Figure 1. Basal and Induced Gene Expression of Relevant OA Genes in Response to IL-1β and TNF-α

(A) qRT-PCR analysis of *IL6*, *IL8*, *IL1β*, *MMP13*, and *ADAMTS5* mRNA levels in non-treated cells and in response to stimulation by IL-1β and TNF-α in chondrocytes from three OA donors. Error bars represent a 95% confidence interval from technical triplicates. (B) Western blot analysis of IL-6 and IL-8 protein levels in non-treated and IL-1β and TNF-α-stimulated conditions in the same donors. β-actin (ACTB) was used as loading control.

(NF-κB)-pathway.[28] miR-140-5p also promotes autophagy in human chondrocytes and other cell types.[29–32] miR-140-3p is known to inhibit TNF-α-induced inflammation in human smooth muscle cells[33] and NF-κB activity in hepatocytes.[34] miR-146a is one of the most studied miRNAs and has been shown to play a central role in immune responses by targeting IRAK1 and TRAF6, two important proteins in the NF-κB cascade.[35,36] miR-146a is upregulated in early OA, possibly to counteract inflammation, and downregulated in late OA.[37] Moreover miR-146a reduced aging-associated and trauma-induced OA by inhibiting Notch 1, IL-1β, and IL-6.[12] Like miR-140-5p, miR-146 promotes autophagy in chondrocytes.[13,38] All this taken into consideration, these three microRNAs are potential candidates for OA gene therapy. Therefore, the current study aimed to further unravel the function of miR-140-5p, miR-140-3p, and miR-146a in an IL-1β and TNF-α induced *in vitro* model of OA. Here we show how single transfection of each miRNA regulated different proteins, often associated with the same biological pathways. miR-140-5p, miR-140-3p, and miR-146a all inhibited inflammation and altered various proteins involved in autophagy, proteasomal and lysosomal degradation pathway, metabolism, and regulation of reactive oxygen species (ROS). miR-140-5p and miR-140-3p also promoted expression of chondrogenic proteins. This supports our hypothesis that these miRNAs are promising candidates in miRNA-based therapy of OA.

RESULTS

Simulating OA *in vitro* using the inflammatory cytokines IL-1β and TNF-α strongly induces the expression of the inflammatory interleukins *IL6*, *IL8*, and *IL1β* and the martix degrading enzyme *MMP13*. Figure 1A shows basal mRNA levels of *IL6*, *IL8*, *IL1β*, *MMP13*, and *ADAMTS5* in non-treated cells and in response to stimulation by IL-1β and TNF-α. The experiment was carried out in three different donors. Figure 1B shows the induced protein expression of IL-6 and IL-8 in response to IL-1β and TNF-α stimulation.

Each miRNA was transfected separately in independent reactions. Successful delivery of the miRNAs was validated with qRT-PCR in all three donors. The miRNAs were detected at much higher levels compared with cells transfected with a negative control sequence (Figure 2A). Figure 2B shows downregulation of RALA and TRAF6 proteins after transfection of miR-140-5p and miR-146a, respectively. RALA and TRAF6 are validated targets of these two miRNAs and showed that the transfected miRNAs were functionally active, although there were varying degrees of knockdown in the different donors. There is no validated target for miR-140-3p in chondrocytes yet.

miR-140-5p, miR-140-3p, and miR-146a Strongly Inhibited IL-1β- and TNF-α-Induced Inflammation

Since all three miRNAs have been shown to inhibit inflammation in different *in vitro* settings, we decided to see if this was also true for our established *in vitro* model of OA. Four days after miRNA transfection, the cells were stimulated with IL-1β and TNF-α. The following day, the cells were harvested. Each of the miRNAs seemed to counteract the inflammatory-mediated expression of *IL6*, *IL8*, and *IL1β* on the mRNA level (Figure 3A). Additionally, each miRNA also

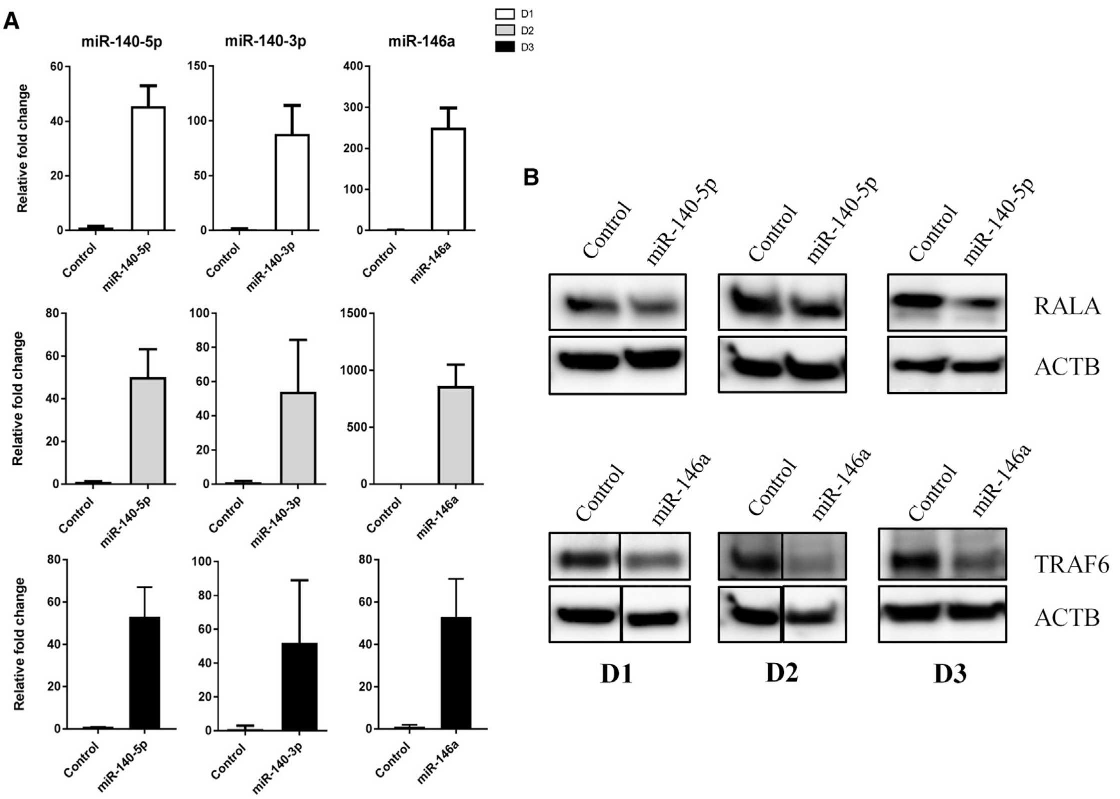

Figure 2. Validation of Increased Levels of miRNAs following Transfection

(A) qRT-PCR analysis of miR-140-5p, miR-140-3p, and miR-146a mRNA levels in chondrocytes from three donors after transfection. Error bars represent a 95% confidence interval from technical triplicates. (B) Western blot analysis of RALA and TRAF6 validated direct targets of miR-140-5p and miR-146a, respectively, in three donors. ACTB was used as loading control.

downregulated IL-6 and IL-8 on the protein level but with varying degrees, depending on donor and miRNA. miR-140-3p and miR-146a exhibited the most potent protective effects, followed by miR-140-5p, with the best effect seen in donor 2 (Figure 3B). miR-140-3p and miR-146a strongly and consistently downregulated *MMP13* in all three donors, while *ADAMTS5* mRNA was downregulated by all three miRNAs in donor 1 cells only (Figure 3A). For technical reasons, donor 3 cells were transfected with miR-140-5p and miR-140-3p one passage after the transfection of miR-146a, necessitating different control samples and thus additional controls in both the qRT-PCR data (Figure 3A) and western blot images (Figure 3B).

Proteome Alteration following Overexpression of the miRNAs under OA-Simulating Conditions

To unravel other important effects these miRNAs might exhibit under the OA-simulated milieu, mass spectrometry proteomics was performed on cell lysates from all three donors. miR-140-5p, miR-140-3p, and miR-146a significantly altered the expression of 40, 36, and 37 proteins, respectively. However, many of the proteins belonged to the same biological pathways (Tables 1, 2, and 3), and some proteins were shared between the miRNAs (Table S1). 36% of the downregulated proteins were predicted to be targeted by miR-140-5p, 12% by miR-140-3p, and 26% by miR-146a (Tables 1, 2, and 3). In addition to inflammation and immune response, proteins involved in autophagy, ER-Golgi transport, the ubiquitin-proteaosomal degradation pathway, ROS regulation, oxidative stress, and metabolism were altered. Cytoskeleton, mRNA/DNA processing, nuclear, and cell cycle control proteins were also altered (Tables 1, 2, and 3). Some selected proteins from the tables are pointed out in the following text. OAS2, IRF9, M4K4, IKIP, and STAT3 were upregulated by miR-140-5p. These proteins are known to be involved in immune responses and regulate inflammation. OAS2 and IRF9 are induced by interferons and inhibit viral replication,[39,40] while M4K4 is a map kinase known to regulate inflammation.[41] IKIP, inhibitor of the NF-κB kinase, is involved in NF-κB regulation, while STAT3 is known to have anti-inflammatory effects.[42] STAT3 is also important in skeletal development and chondrogenesis.[43,44] miR-140-5p also upregulated proteins that are important in maintaining cellular homeostasis processes, such as GBRAP, an autophagy marker that is essential for autophagosome formation, and DHC24, which protects cells against oxidative stress and apoptosis.[45] WNT5A, a transcriptional factor that plays an essential role in chondrocyte differentiation during development through induction of expression of SOX9,[46] was also upregulated by miR-140-5p together

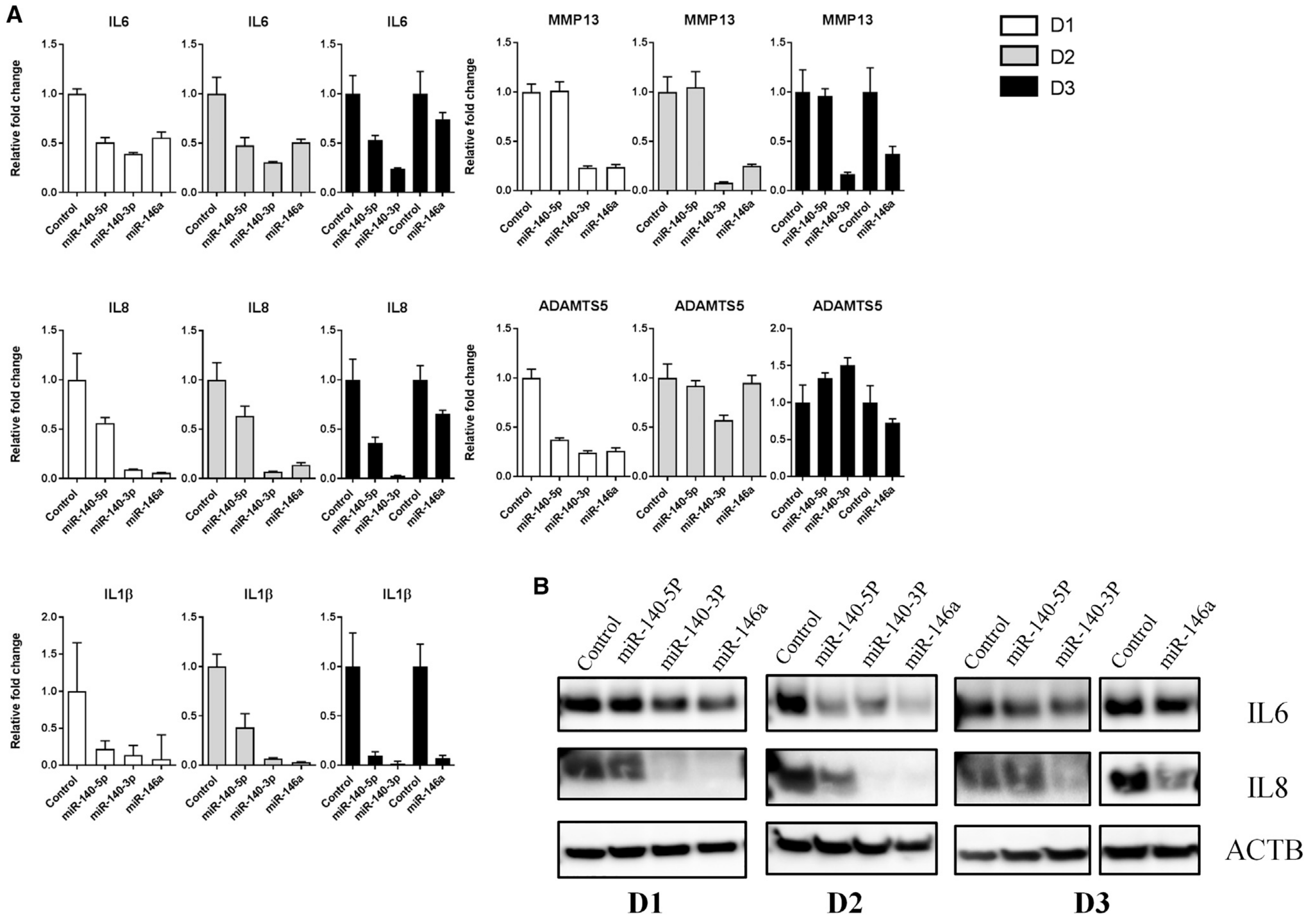

Figure 3. miR-140-5p, miR-140-3p, and miR-146a Counteract IL-1β- and TNF-α-Induced Inflammation

(A) qRT-PCR analysis of *IL6*, *IL8*, *IL1β*, *MMP13*, and *ADAMTS5* mRNA levels in response to overexpression of the three miRs followed by 24 h stimulation with IL-1β and TNF-α in chondrocytes from three donors. Error bars represent a 95% confidence interval from technical triplicates. (B) Western blot analysis of IL-6 and IL-8 in the same three donors. ACTB was used as loading control.

with several proteins involved in intracellular trafficking, histone modification, and other nuclear proteins (Table 1). miR-140-5p downregulated proteins involved in immune responses and some proteins that have undesirable effects on chondrogenesis and OA development, such as STA5A,[47] C1R, and STAM2.[48] STA5A has also been associated with chondrocyte hypertrophy[49] and chondrocyte growth arrest in cartilage of dwarf children.[50,51] C1R, on the other hand, has recently been shown to be upregulated in the synovial fluid of OA patients[52] and OA porcine models.[53] miR-140-5p also downregulated NUP93 and MEP50. The latter has been shown to regulate PRMT5, which is important in maintaining chondro-progenitor cells in mice limb buds.[54] Metabolic enzymes and mitochondrial proteins like ACSL4,[55] APLP2,[56] and PGM2[57] were also downregulated. In addition, several nuclear proteins were downregulated, including HCFC1 and WDR5. The former is involved in cell cycle control, activation, and repression of transcription and has been shown to be involved in craniofacial development in zebrafish,[58] while the latter is involved in histone modifications and is required for osteoblast differentiation.[59] As expected, the direct target of miR-140-5p, RALA, was also downregulated. This is consistent with the western blot results in Figure 2B.

miR-140-3p, like miR-140-5p, upregulated STAT3 and downregulated STA5A, CIR, NUP93, and MEP50 (Tables 2 and S1). In addition, the NF-κB inhibitor IASPP,[60] PDCD4, a tumor suppressor that is downregulated by inflammation,[61] PCOC1, an important enzyme for collagen fibril formation,[62] and DDAH1, an enzyme that reduces oxidative stress,[63] were upregulated. Other upregulated proteins were mainly involved in mitochondria, protein degradation, trafficking, and gene expression processes. LTOR5, an activator of the potent autophagy inhibitor mTORC1, was downregulated by miR-140-3p together with other proteins involved in mitochondria, mRNA processing, and membrane bending.

miR-146a upregulated several proteins involved in the ubiquitination degradation pathway, mitochondrial metabolism, enzymatic cleavage

Table 1. Altered Proteins upon miR-140-5p Overexpression

Protein	Protein Name	Fold Change	Biological Process	Predicted Targets
Uregulated by miR-140-5p				
OAS2	2′-5′-oligoadenylate synthase 2	5.0	inflammation, immune responses, apoptosis	
IRF9	interferon regulatory factor 9	3.0		
M4K4	mitogen-activated protein kinase kinase kinase kinase 4	2.6		
IKIP	inhibitor of nuclear factor kappa-B kinase	2.3		
STAT3	signal transducer and activator of transcription 3	2.3		
GBRAP	gamma-aminobutyric acid receptor-associated protein	5.0	autophagy	
DHC24	delta(24)-sterol reductase	7.0	metabolism, oxidative stress protection	
MVD1	diphosphomevalonate decarboxylase	5.0		
PGM2	phosphoglucomutase-2	2.5		
COG5	conserved oligomeric Golgi complex subunit 5	INF[a]	Golgi apparatus, intracellular vesicle trafficking, cytoskeleton, chaperone	
CKAP5	cytoskeleton-associated protein 5	3.3		
RB3GP	Rab3 GTPase-activating protein catalytic subunit	2.8		
PFD6	prefoldin subunit 6	2.8		
TM9S2	transmembrane 9 superfamily member 2	2.2		
NUP93	nuclear pore complex protein	7.0	nuclear proteins, mRNA processing, spliceosome	
MEP50	methylosome protein 50	6.0		
THOC5	THO complex subunit 5 homolog	INF[a]		
RFOX1	RNA binding protein fox-1 homolog 1	3.0		
TXN4A	thioredoxin-like protein 4A	INF[a]		
WNT5A	protein Wnt-5a	6.0	chondrogenesis	
STRN	Striatin	INF[a]	estrogen and IP3 signaling	
Downregulated by miR-140-5p				
STA5A	signal transducer and activator of transcription 5A	−INF[b]	inflammation	
C1R	complement C1r	−3.0		C1R[c,d]
STAM2	signal transducing adaptor molecule	−INF[b]		
TIM9	mitochondrial import inner membrane translocase subunit Tim9	−3,5	metabolism	
ACSL4	long-chain-fatty-acid-CoA ligase 4	−2.5		
MMSA	methylmalonate-semialdehyde dehydrogenase	−2.1		MMSA[e]
MTDC	bifunctional methylene tetrahydrofolate dehydrogenase	−2.0		MTDC[e]
DCTN3	dynactin subunit 3	−8.0	ER-Golgi transport, intracellular and membrane trafficking, cytoskeleton	
ANFY1	Rabankyrin-5	−4.6		ANFY1[c,d,e,f]
STX4	Syntaxin-4	−INF[b]		
RALA	Ras-related protein Ral-A	−3.3		RALA[c,d,f]
BAG2	BAG family molecular chaperone regulator 2	−2.8		BAG2[c,d]
E41L2	Band 4.1-like protein 2	−2.3		E41L2[c,d]
HCFC1	host cell factor 1	−6.0	nuclear proteins, histone modifications, cell cycle control	
PCNP	PEST proteolytic signal-containing nuclear protein	−INF[b]		
WDR5	WD repeat-containing protein 5	−INF[b]		
LEMD2	LEM domain-containing protein 2	−2.3		

(*Continued on next page*)

Table 1. *Continued*

Protein	Protein Name	Fold Change	Biological Process	Predicted Targets
CSTF2	cleavage stimulation factor subunit 2	−INF[b]	RNA polymerase activity, mRNA splicing	
RPAC1	DNA-directed RNA polymerases I and III subunit	−INF[b]		

INF, infinity.
[a]Only detected in miRNA transfected cells
[b]Only detected in control transfected cells
[c]Predicted targets according to TargetScan
[d]Predicted targets according to miRDB
[e]Predicted target according to miRwalk: prediction based on coding region
[f]Predicted target according to miRwalk: prediction based on 3′UTR

of procollagens (PCOC1, shared with miR-140-3p), and GTPase activation activity. miR-146a additionally downregulated several proteins involved in immune responses and inflammation, including the NF-κB activators RIPK2,[64] TAP1,[65] SEP10,[66] and STAT2.[67] miR-146a also led to downregulation of proteins involved in ROS generation, like the pro-apoptotic QORX.[68] IL-6, IL-8, and TRAF6, all detected by western blot, were not detected by the mass spectrometry analysis and are therefore not included in the lists of differently expressed proteins.

Validation of Proteomics Findings: Selected Genes

miR-140-5p Transfected Cells

GBRAP was validated by western blot and showed consistent upregulation in all three donors (Figure 4A). However, the mRNA level was only upregulated in donor 1, showing mRNA-protein discrepancy (Figure 4B).[69] Moreover, detection of autophagic flux through inhibition of lysosomal degradation using Bafilomycin A1 resulted in accumulation of lipidated GBRAP (GBRAPII) (Figure 4C), which would have been degraded by autophagy. The accumulation of GBRAPII provides an estimate of the autophagic activity. *CIR* mRNA were downregulated in all donors, while *STAT5A* mRNA was downregulated in two of the donors (Figure 4D). *RALA* mRNA, a validated direct target of miR-140-5p, showed consistent downregulation in all three donors (Figure 4D) and by western blot (Figure 2B). DHCR24, the gene coding for DHC24, showed upregulation on mRNA levels in all three donors (Figure 4D).

miR-140-3p Transfected Cells

Due to limited material from the miR-140-3p transfected cells, the western blot validation experiment was repeated in a fourth donor. The proteins LTOR5 and PDCD4 showed the same expression pattern as the proteomics for all three donors (Figure 4E). *LAMTOR5* mRNA, coding for LTOR5, on the other hand, was downregulated in donor1 only (Figure 4F). CIR and STAT5A were also targeted by miR-140-3p and were downregulated at mRNA levels, except for *CIR* in donor 3 (Figure 4G). BROX, a predicted target of miR-140-3p, was downregulated at the mRNA levels in two donors, while mRNA for *DDAH1* showed upregulation on the mRNA levels in two donors (Figure 4G).

miR-146a Transfected Cells

RIPK2 was downregulated at both the protein and mRNA levels in all three donors (Figures 4H and 4I). *STAT2* mRNA was downregulated in one donor, while *TP5313* mRNA, the gene coding for QORX, was downregulated in two donors (Figure 4J)

DISCUSSION

The exact causes of OA are unknown, but at the cellular level, inflammation, reduced autophagy, increased production of ROS, increased mitochondrial DNA damage, and altered metabolism are all hallmarks of OA chondrocytes.[70,71] All these processes are linked together in a complex network.[72,73] Inflammation is thought to be a perpetuating force driving disease progression by upregulating enzymes that break down the articular cartilage.[74] Autophagy has been shown to control inflammation by degrading IL-1β and inhibiting its secretion, while inhibition of autophagy enhanced processing and secretion of IL-1β. Moreover, this effect was suggested to be reduced by ROS inhibition.[73,75] ROS itself can lead to mitochondrial DNA damage and altered metabolism.[76,77] Thus, changes in one of the processes will likely affect the other processes.

miRNAs are known to affect many genes belonging to the same biological process.[78–81] miRNA-based gene therapy strategies are therefore promising candidates for the treatment of OA by fine-tuning or ensuring homeostatic control of some of the cellular processes that are altered in OA. miR-140-5p, miR-140-3p, and miR-146a are all involved in cartilage and OA biology and are potential candidates for miRNA-based therapy of OA. However, there is much to learn about their functional roles in cartilage and OA. Here we show how these miRNAs act on several cellular pathways associated with OA in an IL-1β and TNF-α induced *in vitro* model of OA, including inflammation, autophagy, ROS regulation, and metabolism. Other proteins involved in the ubiqutin degradation pathway, ER-Golgi-trafficking, mRNA processing, and cell cycle control, were also altered by these miRNAs.

Our initial results showed upregulation of OA genes and proteins in chondrocytes in response to IL-1β and TNF-α. To address our hypothesis about the protective effects of the three miRs under these conditions, we first validated their successful overexpression in all donors, yet with a variation that is likely to be caused by natural donor variation. The miRNAs led to downregulation of *IL6*, *IL8*, and *IL1β* on the mRNA level as well as on the protein level, with variation in the degree of potency. Overall miR-140-3p and miR-146a gave better

Table 2. Altered Proteins upon miR-140-3p Overexpression

Protein	Protein Name	Fold Change	Biological Process	Predicted Targets
Upregulated by miR-140-3p				
ISAPP	RelA-associated inhibitor	INF[a]	inflammation, immune response, cell growth, apoptosis	
GILT	gamma-interferon-inducible lysosomal thiol reductase	INF[a]		
CNPY4	protein canopy homolog 4	2.8		
STAT3	signal transducer and activator of transcription 3	2.1		
DDAH1	N(G),N(G)-dimethylarginine dimethylaminohydrolase 1	INF[a]	mitochondrial respiratory machinery, NOS/ROS regulation	
NDUS7	NADH dehydrogenase [ubiquinone] iron-sulfur protein 7, mitochondrial	3.1		
PPIL3<br>DSS1	peptidyl-prolyl cis-trans isomerase-like 3<br>26S proteasome complex subunit DSS1	5.2<br>INF[a]	proteasome, immunoproteasome, chaperones	
PSMG2	proteasome assembly chaperone 2	INF[a]		
PSMD9	26S proteasome non-ATPase regulatory subunit 9	INF[a]		
ZFPL1	zinc finger protein-like 1	INF[a]	ER-protein, Golgi, vesicle trafficking	
COG3	conserved oligomeric Golgi complex subunit 3	INF[a]		
VP37C	vacuolar protein sorting-associated protein 37C	2.8		
ERLEC	endoplasmic reticulum lectin 1	2.0		
PDCD4	programmed cell death protein 4	10.0	tumor suppressor, apoptosis	
IBP4	insulin-like growth factor-binding protein 4	INF[a]		
PP4R1	serine/threonine-protein phosphatase 4 regulatory subunit 1	INF[a]	chromatin, histone modifications, nuclear, mRNA export from the nucleus	
NUP93	nuclear pore complex protein Nup93	7.2		
MEP50	methylosome protein 50	5.1	splisosome, transcription regulation, mRNA/DNA processing	
RNH2A	ribonuclease H2 subunit A	INF[a]		
T2AG	transcription initiation factor IIA subunit 2	INF[a]		
MYOV2	myeloma-overexpressed gene 2 protein	2.0		
PININ	Pinin	2.0		
PP12C	protein phosphatase 1 regulatory subunit 12C	INF[a]	scaffold protein, actin cytoskeleton	
NHRF1	Na(+)/H(+) exchange regulatory cofactor NHE-RF1	2.0		
FSTL1	follistatin-related protein 1	2.2	skeletal developement	
PCOC1	procollagen C-endopeptidase enhancer 1	2.1	glycoprotein that binds and drives enzymatic cleavage of type I procollagen	
Downegulated by miR-140-3p				
STA5A	signal transducer and activator of transcription 5A	−5.0	inflammation, innate immunity	
C1R	complement C1r subcomponent	−2.0		
LTOR5	regulator complex protein LAMTOR5	−2.3	autophagy	
MIC27	MICOS complex subunit MIC27	−5.0	mitochondrial proteins and chaperopnes	
RT35	28S ribosomal protein S35, mitochondrial	−INF[b]		
GPDM	glycerol-3-phosphate dehydrogenase, mitochondrial	−2.1		
CSTF1	cleavage stimulation factor subunit 1	−3.0	mRNA processing	
BROX	BRO1 domain-containing protein	−3.0	membrane bending	BROX[c]

INF, infinity.
[a]Only detected in miRNA transfected cells
[b]Only detected in control transfected cells
[c]Predicted target according to miRwalk: prediction based on 3′UTR

Table 3. Altered Proteins upon miR-146a Overexpression

Protein	Protein Name	Fold Change	Biological Process	Predicted Targets
Upregulated by miR-146a				
CUL1	Cullin-1	7.0	ubiquitination and degredation, lysosomal	
NEUR1	Sialidase-1	6.0		
PPIF	peptidyl-prolyl cis-trans isomerase F, mitochondrial	3.8	mitochondrial metabolism, phospholipid metabolism	
PCAT1	lysophosphatidylcholine acyltransferase	2.6		
PCOC1	procollagen C-endopeptidase enhancer 1	2.0	enzymatic cleavage of type I procollagen	
GIT2	ARF GTPase-activating protein GIT2	2.3	GTPase-activating protein (GAP) activity	
Downregulated by miR-146a				
TAP1	antigen peptide transporter 1	−INF[a]	inflammation, innate/adaptive immune responses	
SEP10	Septin-10	−7.0		
STAT2	signal transducer and activator of transcription 2	−4.4		STAT2[b,c]
RIPK2	receptor-interacting serine/threonine-protein kinase 2	−INF[a]		
ABCF1	ATP-binding cassette sub-family F member 1	−3.3		
SHPK	sedoheptulokinase OS	−INF[a]		SHPK[b,c]
QORX	quinone oxidoreductase PIG3	−4.5	oxidative stress/ROS	
MT1E	Metallothionein-1E	−3.0		
LAMP1	lysosome-associated membrane glycoprotein 1 (CD107a)	−3.0	lysosomal, chaperone/ proteinfolding	
DNJA1	DnaJ homolog subfamily A member 1	−2.8		
HYOU1	hypoxia upregulated protein 1	−2.3		HYOU1[c]
NDUA2	NADH dehydrogenase [ubiquinone] 1 alpha subcomplex subunit 2	−INF[a]	mitochondrial respiratory, metabolism	
TOM34	mitochondrial import receptor subunit	−5.0		
F120B	constitutive coactivator of peroxisome proliferator-activated receptor gamma	−INF[a]		F120B[c,d]
PNPO	pyridoxine-5′-phosphate oxidase	−2.7		
HCFC1	host cell factor 1	−6.0	transcription and cell cycle control, histone, chromatin factors, and nuclear proteins	
SP16H	FACT complex subunit SPT16	−6.0		
PELO	protein pelota homolog	−INF[a]		PELO[d]
DDX21	nucleolar RNA helicase 2	−2.2		
AAAS	Aladin	−2.0		
GTPB1	GTP-binding protein 1	−INF[a]	degradation of target mRNA, circadian mRNA stability	
MED18	mediator of RNA polymerase II transcription subunit 18	−INF[a]	coactivator of transcripton of all RNA pol II genes	
TM109	transmembrane protein 109	−INF[a]	DNA-damage response/DNA repair	
TRIPC	E3 ubiquitin-protein ligase	−INF[a]		
ADPPT	L-aminoadipate-semialdehyde dehydrogenase-phosphopantetheinyl transferase	−INF[a]	post-translational modification	
ATAD1	ATPase family AAA domain-containing protein 1	−INF[a]	regulation of cell surface expression of AMPA receptors	
FMNL3	formin-like protein 3	−INF[a]	cytoskeletal organization and adherens juctions	FMNL3[b]
GEPH	Gephyrin	−INF[a]		GEPH[c]
VEZA	Vezatin	−INF3[a]		

(*Continued on next page*)

Table 3. *Continued*

Protein	Protein Name	Fold Change	Biological Process	Predicted Targets
RAGP1	ran GTPase-activating protein 1	−2.2	trafficking, transport from the cytoplasm to the nucleus	
CRK	adaptor molecule crk (p38)	−2.2	proto-oncogene, several signaling pathways	CRK[e]

INF, infinity.
[a]Only detected in control transfected cells
[b]Predicted targets according to miRwalk: prediction based on 3′UTR
[c]Predicted targets according to miRwalk: prediction based on coding region
[d]Predicted targets according to miRwalk: prediction based on 5′UTR
[e]Predicted targets according to miRDB

reduction of *IL6* and *IL8* compared with miR-140-5p. miR-140-3p and miR-146a also exhibited a strong downregulatory effect on *MMP13*. *ADAMTS5* mRNA was downregulated by all three miRNAs in one donor and by miR-140-3p in another donor.

Proteomic analysis revealed a broader picture of how these miRNAs might work. The three miRNAs downregulated different pro-inflammatory mediators. miR-140-5p and miR-3p both downregulated STA5A and C1R, two proteins that have been associated with inflammation. STA5A was detected as an inflammatory response biomarker[47] and has been associated with chondrocyte hypertrophy in mice[49] and dwarfism in humans.[51] STA5A has also been shown to have a binding site in the *ADAMTS5* promoter.[82] C1R is the first component of the complement system and has been shown to be upregulated in the synovial fluid of OA patients.[52] C1R activates C1S, which has been implicated in matrix degradation of articular cartilage in rheumatoid arthritis (RA).[83] Additionally miR-140-5p and miR-140-3p upregulated the anti-inflammatory mediator STAT3, which is also important for SOX9 expression and cartilage formation during development.[44] ISAPP, a potent inhibitor of NF-κB, was upregulated by miR-140-3p. miR-146, on the other hand, downregulated inflammation and immune-related proteins: TAP1, SEP10, STAT2, ABCF1, and RIPK2. TAP1 is an antigen peptide transporter upregulated by TNF-α in chondrocytes[84] and is known to play an important role in immune responses, with a polymorphism that has recently been shown to be associated with the inflammatory joint disease ankylosing apondylitis.[85] SEP10 is induced by TNF-α.[86] STAT2 was detected in OA chondrocytes but not in healthy controls, suggesting a role in OA.[87] ABCF1 is thought to be a regulator of the translation of inflammatory cytokine pathways, and it has been shown to regulate and be regulated by TNF-α.[88–90] RIPK2 was validated to be strongly downregulated on both the mRNA and protein level. RIPK2 is an upstream activator of NF-κB and plays an essential role in modulating innate and adaptive immune responses.[64,91] A hyperactive RIPK2 allele is involved in onset of early OA.[92] RIPK2 has a distinct expression profile, together with cartilage destruction markers in chondrocytes stimulated with synovial fibroblasts from RA patients.[93] RIPK2 downregulation also inhibited catabolic genes induced by cartilage damaging toxin T-2.[94] Thus, our results support the *in vivo* findings that miR-146a inhibited OA development. In summary, all three miRNAs have an overall inhibitory effect on inflammation, which is likely to be beneficial for the prevention of OA.

The role of miR-146a as an anti-inflammatory miRNA has been extensively elucidated in the literature, and its roles as age and OA-attenuating, cartilage protecting,[12] and autophagy enhancing[38] are emerging. Yet one study published last year by Zhang et al.[95] claimed a contradicting role to miR-146a in mice. In this study, the authors show that miR-146a KO mice have less cartilage degeneration compared to WT mice in spontaneous and instability induced OA models. They also show that miR-146a aggravates pro-inflammatory cytokines and suppresses the expression of COL2A and SOX9. Whether that could be explained by how the KO mice were generated or other factors is yet to be understood.

Autophagy is an essential mechanism that ensures cellular homeostasis by degrading old and damaged cellular components and recycling of macromolecules. The consequence of reduced autophagy and other degradation pathways is production of ROS, which may lead to DNA damage and ultimately cell death.[8,96] Reduced autophagy, accumulation of dysfunctional organelles and/or proteins, and increased ROS production has been reported in OA chondrocytes in several studies,[97–101] while enhancing autophagy was shown to be chondro-protective in a mouse model of OA.[99] This suggests that altered autophagy is involved in OA development. Our data showed a pro-autophagy tendency for all three miRNAs. GBRAP, an autoghagy marker and a member of the Autophagy-related protein8 (ATG8) family, which is crucial for autophagosome formation and degradation of cytosolic cargo, was upregulated by miR-140-5p. We detected autophagic flux by western blot, evident by the conversion of GBRAP from the type I to type II form involved in autophagosome biogenesis. miR-140-5p led to more accumulation of GBRAPII compared to control. However, when stimulated with cytokines, the effect was reduced. A pro-autophagic role of miR-140-5p is consistent with previous findings where miR-140-5p promoted autophagy in human chondrocytes.[29] A pro-autophagic effect of miR-140-5p has also been demonstrated in other cell types.[30–32]

miR-140-3p, on the other hand, downregulated the autophagy inhibitory protein LTOR5 (encoded by the gene *LAMTOR5*). Autophagy is closely linked with the ER-Golgi and proteasomal

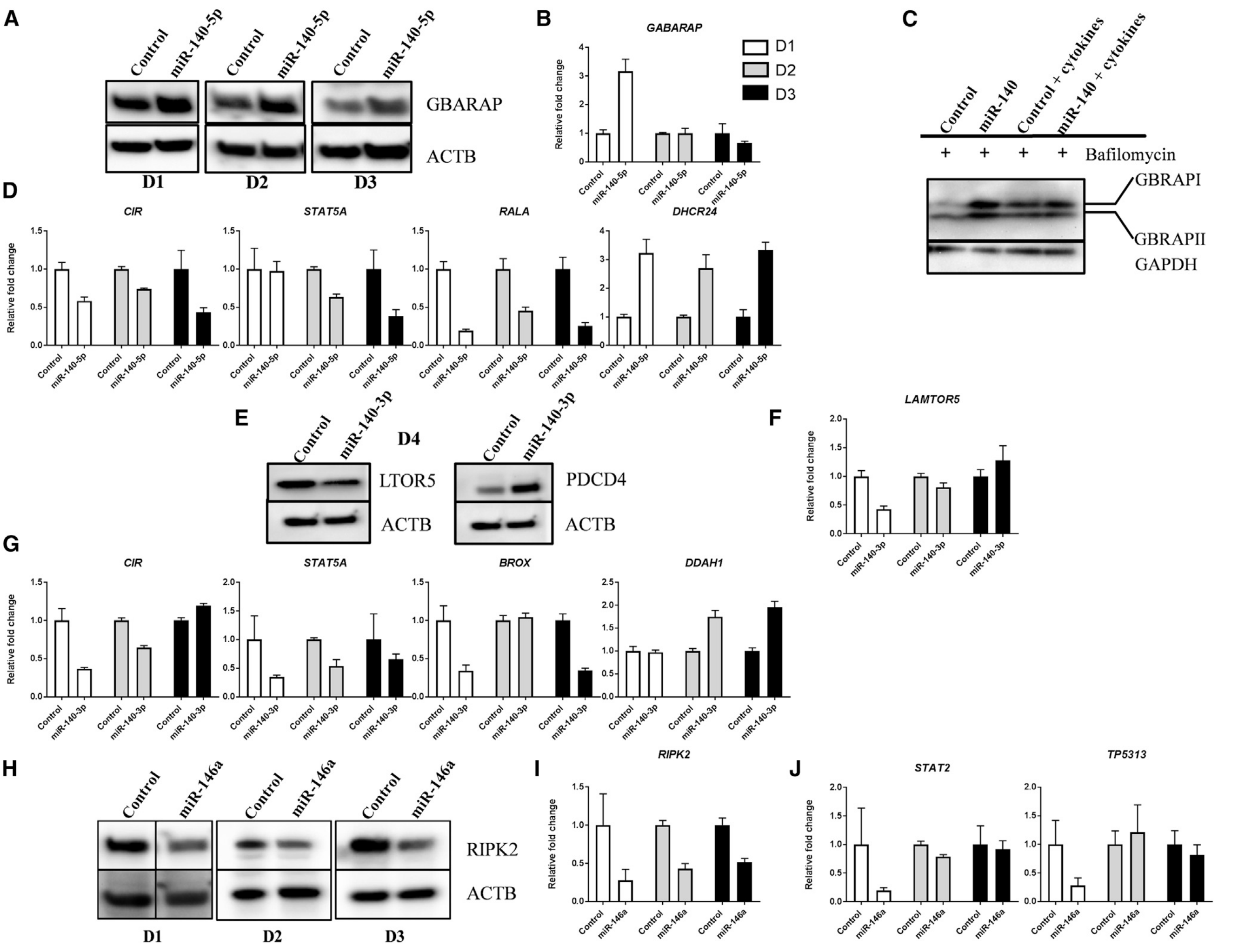

Figure 4. Validation of Proteomics Results by qRT-PCR and Western Blot Analysis

(A) GBRAP protein levels: ACTB was used as loading control. (B) *GABARAP* mRNA levels and (C) GBRAPI and -II after treatment with Bafilomycin A1. GAPDH was used as loading control. (D) *CIR*, *STAT5A*, *RALA*, and *DHCR24* mRNA levels after overexpression of miR-140-5p in three donors. (E) LTOR5 and PDCD4 protein levels after overexpression of miR-140-3p in a fourth donor. (F) *LAMTOR5* mRNA levels and (G) *CIR*, *STAT2*, *BROX*, and *DDAH1* mRNA levels after overexpression of miR-140-3p in three donors. (H) RIPK2 protein levels, (I) *RIPK2* mRNA levels, and (J) *STAT2* and *TP5313* mRNA levels after overexpression of miR-146a in three donors. ACTB was used as loading control for all western blots. Error bars from qRT-PCR represent a 95% confidence interval from technical triplicates.

degradation systems. miR-140-5p, miR-140-3p, and miR-146a led to both upregulation and downregulation of several proteins involved in these processes, perhaps suggesting a regulatory role to establish homeostatic control. Also, ROS and oxidative stress proteins were affected by the three miRNAs. miR-140-5p upregulated DHC24, an enzyme that protects cells against oxidative stress and apoptosis by reducing caspase 3 activity.[45] Interestingly, this protein is also important in cartilage and skeletal development, as mutations within this gene lead to severe developmental abnormalities, including short limbs.[102] Mirza et al.[103] showed that bones from *DHC24* KO mice lacked proliferating chondrocytes in the growth plate and showed abnormal hypertrophy of prehypertrophic chondrocytes. In addition, H_2O_2-induced hypertrophy was prevented by lentiviral delivery of DHC24. Thus, miR-140-5p might protect the cells from ROS through upregulation of DHC24. DHC24 was validated by qRT-PCR to be upregulated in all three donors. miR-140-3p may protect against oxidative stress by upregulating the enzyme DDAH1. Shi et al.[104] demonstrated that DDAH1 deficiency increased oxidative stress and led to increased kidney fibrosis in mice. DDAH1 mRNA upregulation was validated by qRT-PCR in two donors. QORX, on the other hand, was downregulated by miR-146a. Porte et al.[68] showed that overexpression of QORX accumulated ROS both *in vitro* and *in vivo*. Its downregulation in our data might suggest that miR146a protects chondrocytes from excessive ROS formation. QORX (TP5313) mRNA showed downregulation in two donors.

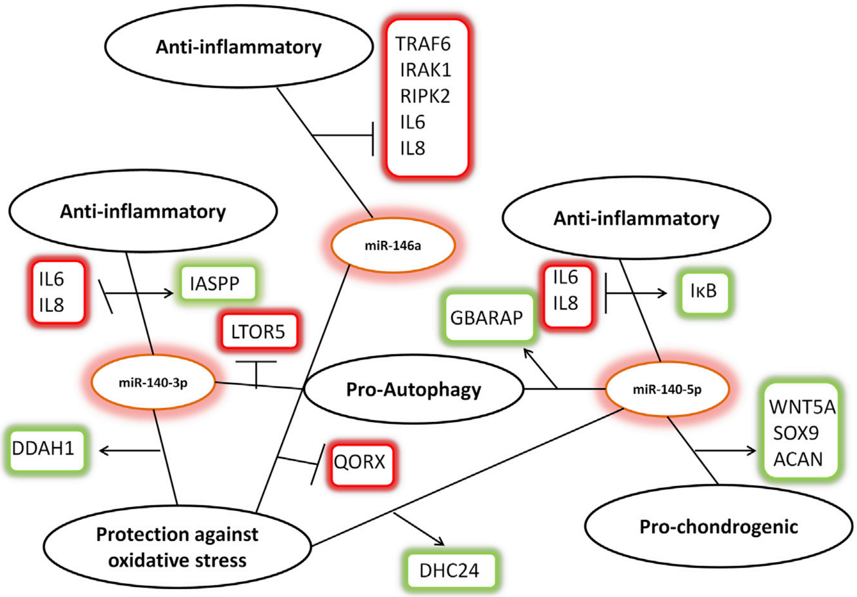

Figure 5. A Proposed Model of the Three miRNAs' Mode of Action

miR140-5p, miR-140-3p, and miR-146a regulated expression of key components of inflammation, autophagy, and other degradation pathways. A proposed model of how this might promote cartilage integrity and protection under adverse inflammatory conditions is shown. Arrows and green boxes represent positive regulation, while the perpendicular lines and red boxes represent inhibition.

Figure 5 illustrates some of the proteomics findings and shows the many possible platforms that can be altered to prevent or perhaps even treat OA by delivery of miR-140-5p, miR-140-3p, and miR-146a. SOX9, ACAN, and IκB, three important proteins that we have shown previously to be positively regulated by miR-140-5p,[26,28] are included.

OA is a multietiological disease and it is necessary to acquire sufficient knowledge about the different processes and their complex interplay in order to provide successful treatment of OA. We demonstrate here that miR-140-5p, miR-140-3p, and miR-146a inhibited IL-1β– and TNF-α-induced inflammation, promoted autophagy, and regulated several proteins involved in ROS production and metabolism. Thus, intraarticular delivery of one, or several, of these miRNAs may be a promising therapeutic option for OA.

MATERIALS AND METHODS

Isolation and Culture of Human Articular Chondrocytes (ACs)

ACs were isolated from discarded OA cartilage tissue after total knee replacement surgery and cultured as previously described.[23] Only tissue with no macroscopic signs of OA was used. All donors provided written informed consent. The study was approved by the Regional Committee for Medical Research Ethics, Southern Norway. Briefly, the cartilage was cut into tiny pieces and subsequently digested with Collagenase type XI (Sigma-Aldrich, St. Louis, MO, USA) at 37°C for 90–120 min. Chondrocytes were washed three times and resuspended in culture medium consisting of DMEM/F12 (GIBCO/ThermoFisher Scientific, Waltham, MA, USA) supplemented with 10% human plasma (Octaplasma AB, Oslo Blood Bank, Norway) supplemented with platelet lysate (corresponding to 10^9 platelets/mL plasma) (PLP), 100 U/mL penicillin, 100 μg/mL streptomycin, and 2.5 μg/mL amphotericin B.[23] PLP was prepared as previously described.[105] The culture medium was changed every 3–4 days. After the first passage, amphotericin B was removed. At 70%–80% confluence, cells were detached with trypsin-EDTA (Sigma-Aldrich) and seeded into new culture flasks.

miRNA Mimics, Transfection, and Stimulation with IL-1β and TNF-α

The Amaxa nucleofector system and the Amaxa Human Chondrocyte Nucleofector Kit were used for electroporation following the protocols from the manufacturer (Lonza, Walkersville, MD, USA). Briefly, each reaction contained 1.0×10^6 cells, 5μM of pre-miR mimics (Table S2) in a total volume of 100 μL nucleofection solution. The cells were seeded in 20% PLP without antibiotics and left to recover overnight. The following day (day 1), the medium was changed to 10% PLP with 1% penicillin/streptomycin. On day 4, ACs were stimulated with 0.1 ng/mL recombinant IL-1β (rIL-1β) or 10 ng/mL rTNF-α (R&D Systems, Minneapolis, MN) for 24 h before harvesting for analysis.

Autphagic Flux

On day 5, 2 h prior to harvesting the cells, ACs were treated with 100 nM Bafilomycin A1 (Sigma-Aldrich).

Isolation of miRNA, cDNA Synthesis, and qRT-PCR

Total RNA containing miRNAs was isolated using the miRNeasy mini kit according to the manufacturer's protocol (QIAGEN, Germantown, MD, USA). cDNA synthesis and qRT-PCR were performed following protocols from the manufacturer using the Taqman MicroRNA Reverse Transcription Kit (Thermo Fisher Scientific, Waltham, MA, USA). 10 ng miRNA in a total volume of 15 μL was reverse transcribed into cDNA. All samples were run in technical triplicates. Each replicate contained 1.33 μL cDNA in a total volume of 15 μL. The thermocycling parameters were 95°C for 10 min, followed by 40 cycles of 95°C for 15 s and 60°C for 1 min. U18 was used as endogenous control. qRT-PCR results are shown as relative fold changes using mean values from technical triplicates with a 95% confidence interval. All donors are shown separately in the figures.

Western Blotting

Cell lysates corresponding to 200,000 cells were loaded onto a 4%–20% gradient or 10% polyacrylamide gel (Bio-Rad, Hercules, CA, USA). Proteins were separated be gel electrophoresis, transferred

to polyvinylidene fluoride or polyvinylidene difluoride (PVDF) membranes and incubated with appropriate antibodies before visualizing the bands using the myECL imager (Thermo Fisher Scientific).

In-Solution Digestion

400 μL ice cold acetone (Sigma-Aldrich, Oslo, Norway) was added to the samples, vortexed, and precipitated at −20°C overnight. Samples were centrifuged at 16,000 *g* for 20 min at 4°C, and the supernatant was discarded. Proteins were re-dissolved in 50 μL 6 M urea and 100 mM ammonium bicarbonate (Sigma-Aldrich), pH 7.8. For reduction and alkylation of cysteines, 2.5 μL 200 mM dithiothreitol (DTT; Sigma-Aldrich) was added, and the samples were incubated at 37°C for 1 h followed by addition of 7.5 μL 200 mM iodoacetamide (Sigma-Aldrich) for 1 h at room temperature in the dark. The alkylation reaction was quenched by adding 10 μL 200 mM DTT. The proteins were digested with trypsin gold (Promega, Madison, WI, USA) in a final volume of 250 μL for 16 h at 37°C. The digestion was stopped by adding 20 μL 1% formic acid (Sigma-Aldrich), and the generated peptides were purified using ZipTips (Millipore, Billerica, MA, USA) and dried using a Speed Vac concentrator (Eppendorf, Hamburg, Germany).

Nano LC-QExactive Orbitrap Mass Spectrometry

Peptides were analyzed using an Ultimate 3000 nano-ultra-high-performance liquid chromatography (UHPLC) system (Dionex, Sunnyvale, CA, USA) connected to a Q Exactive mass spectrometer (ThermoElectron, Bremen, Germany) equipped with a nano electrospray ion source. For liquid chromatography separation, an Acclaim PepMap 100 column (C18, 3 μm beads, 100 Å, 75 μm inner diameter) (Dionex, Sunnyvale CA, USA) capillary of 50 cm bed length was used. A flow rate of 300 nL/min was employed with a solvent gradient starting with 97% solvent A and 3% solvent B (A is always 100% B) to 35% B for 97 min, and to 50% B for 20 min, and then to 80% B for 2 min. Solvent A was 0.1% formic acid (in water) and solvent B was 0.1% formic acid, 90% acetonitrile, and 9.9% water. The mass spectrometer was operated in the data-dependent mode to automatically switch between mass spectrometry (MS) and tandem MS (MS/MS) acquisition. Survey full scan MS spectra (from m/z 400 to 1,700) were acquired with the resolution R = 70,000 at m/z 200 after accumulation to a target of 1e6. The maximum allowed ion accumulation times were 100 ms. The method used allowed sequential isolation of up to the ten-most intense ions, depending on signal intensity (intensity threshold 1.7e4), for fragmentation using higher collision induced dissociation (HCD) at a target value of 10,000 charges and a resolution R = 17,500. Target ions already selected for MS/MS were dynamically excluded for 30 s. The isolation window was m/z = 2 without offset. The maximum allowed ion accumulation for the MS/MS spectrum was 60 ms. For accurate mass measurements, the lock mass option was enabled in MS mode, and the polydimethylcyclosiloxane ions generated in the electrospray process from ambient air were used for internal recalibration during the analysis.

Data Analysis

Data were acquired using Xcalibur v2.5.5 and raw files were processed to generate peak list in Mascot generic format (*.mgf) using ProteoWizard release version 3.0.7230. Database searches were performed using Mascot in-house version 2.4.0 to search the SwissProt database (human, 21.01.2016, 20187 proteins) assuming the digestion enzyme trypsin, at maximum one missed cleavage site, fragment ion mass tolerance of 0.05 Da, parent ion tolerance of 10 ppm, and oxidation of methionines, and acetylation of the protein N terminus as variable modifications. Scaffold (version Scaffold_4.4.3, Proteome Software, Portland, OR) was used to validate MS2-based peptide and protein identifications. Peptide identifications were accepted if they could be established at greater than 95.0% probability by the Scaffold local false discovery rate (FDR) algorithm. Protein identifications were accepted if they could be established at greater than 99.9% probability. A threshold of 2-fold and multiple testing corrections ($p < 0.05$, Benjamini Hochberg) were used for analysis of differently expressed proteins using the Scaffold software.

SUPPLEMENTAL INFORMATION

Supplemental Information can be found online at https://doi.org/10.1016/j.omtn.2019.07.011.

AUTHOR CONTRIBUTIONS

Conceptualization, R.N.A, T.A.K., and J.E.B.; Methodology, R.N.A.; Investigation, R.N.A, T.A.K., and J.E.B.; Writing – Original Draft, R.N.A.; Writing – Review & Editing, T.A.K. and J.E.B.; Funding Acquisition, J.E.B.; Resources, J.E.B.; Supervision, T.A.K. and J.E.B.

CONFLICTS OF INTEREST

The authors declare no competing interests.

ACKNOWLEDGMENTS

Proteomics services were purchased from the Proteomics Core Facility, University of Oslo, and performed by Bernd Thiede and Christian Koehler. We would also like to acknowledge Dr. Bjorn Ødegaard for providing cartilage tissue samples. This study was funded by a PhD grant from South-Eastern Norway Regional Health Authority.

REFERENCES

1. Heidari, B. (2011). Knee osteoarthritis prevalence, risk factors, pathogenesis and features: Part I. Caspian J. Intern. Med. *2*, 205–212.
2. Losina, E., Walensky, R.P., Reichmann, W.M., Holt, H.L., Gerlovin, H., Solomon, D.H., Jordan, J.M., Hunter, D.J., Suter, L.G., Weinstein, A.M., et al. (2011). Impact of obesity and knee osteoarthritis on morbidity and mortality in older Americans. Ann. Intern. Med. *154*, 217–226.
3. Lotz, M.K., and Caramés, B. (2011). Autophagy and cartilage homeostasis mechanisms in joint health, aging and OA. Nat. Rev. Rheumatol. *7*, 579–587.
4. Sellam, J., and Berenbaum, F. (2010). The role of synovitis in pathophysiology and clinical symptoms of osteoarthritis. Nat. Rev. Rheumatol. *6*, 625–635.
5. Goldring, M.B., and Otero, M. (2011). Inflammation in osteoarthritis. Curr. Opin. Rheumatol. *23*, 471–478.
6. Mengshol, J.A., Vincenti, M.P., Coon, C.I., Barchowsky, A., and Brinckerhoff, C.E. (2000). Interleukin-1 induction of collagenase 3 (matrix metalloproteinase 13) gene expression in chondrocytes requires p38, c-Jun N-terminal kinase, and nuclear factor kappaB: differential regulation of collagenase 1 and collagenase 3. Arthritis Rheum. *43*, 801–811.
7. Hashimoto, M., Nakasa, T., Hikata, T., and Asahara, H. (2008). Molecular network of cartilage homeostasis and osteoarthritis. Med. Res. Rev. *28*, 464–481.

8. Caramés, B., Taniguchi, N., Otsuki, S., Blanco, F.J., and Lotz, M. (2010). Autophagy is a protective mechanism in normal cartilage, and its aging-related loss is linked with cell death and osteoarthritis. Arthritis Rheum. *62*, 791–801.

9. Hui, W., Young, D.A., Rowan, A.D., Xu, X., Cawston, T.E., and Proctor, C.J. (2016). Oxidative changes and signalling pathways are pivotal in initiating age-related changes in articular cartilage. Ann. Rheum. Dis. *75*, 449–458.

10. Yan, S., Wang, M., Zhao, J., Zhang, H., Zhou, C., Jin, L., Zhang, Y., Qiu, X., Ma, B., and Fan, Q. (2016). MicroRNA-34a affects chondrocyte apoptosis and proliferation by targeting the SIRT1/p53 signaling pathway during the pathogenesis of osteoarthritis. Int. J. Mol. Med. *38*, 201–209.

11. Park, K.W., Lee, K.-M., Yoon, D.S., Park, K.H., Choi, W.J., Lee, J.W., and Kim, S.H. (2016). Inhibition of microRNA-449a prevents IL-1β-induced cartilage destruction via SIRT1. Osteoarthritis Cartilage *24*, 2153–2161.

12. Guan, Y.-J., Li, J., Yang, X., Du, S., Ding, J., Gao, Y., Zhang, Y., Yang, K., and Chen, Q. (2018). Evidence that miR-146a attenuates aging- and trauma-induced osteoarthritis by inhibiting Notch1, IL-6, and IL-1 mediated catabolism. Aging Cell *17*, e12752.

13. D'Adamo, S., Cetrullo, S., Minguzzi, M., Silvestri, Y., Borzì, R.M., and Flamigni, F. (2017). MicroRNAs and Autophagy: Fine Players in the Control of Chondrocyte Homeostatic Activities in Osteoarthritis. Oxid. Med. Cell. Longev. *2017*, 3720128.

14. Lee, R.C., Feinbaum, R.L., and Ambros, V. (1993). The C. elegans heterochronic gene lin-4 encodes small RNAs with antisense complementarity to lin-14. Cell *75*, 843–854.

15. Meijer, H.A., Smith, E.M., and Bushell, M. (2014). Regulation of miRNA strand selection: follow the leader? Biochem. Soc. Trans. *42*, 1135–1140.

16. Castanotto, D., Sakurai, K., Lingeman, R., Li, H., Shively, L., Aagaard, L., Soifer, H., Gatignol, A., Riggs, A., and Rossi, J.J. (2007). Combinatorial delivery of small interfering RNAs reduces RNAi efficacy by selective incorporation into RISC. Nucleic Acids Res. *35*, 5154–5164.

17. Lim, L.P., Lau, N.C., Garrett-Engele, P., Grimson, A., Schelter, J.M., Castle, J., Bartel, D.P., Linsley, P.S., and Johnson, J.M. (2005). Microarray analysis shows that some microRNAs downregulate large numbers of target mRNAs. Nature *433*, 769–773.

18. Xu, Q., Seeger, F.H., Castillo, J., Iekushi, K., Boon, R.A., Farcas, R., Manavski, Y., Li, Y.G., Assmus, B., Zeiher, A.M., and Dimmeler, S. (2012). Micro-RNA-34a contributes to the impaired function of bone marrow-derived mononuclear cells from patients with cardiovascular disease. J. Am. Coll. Cardiol. *59*, 2107–2117.

19. Liu, C.-J. (2009). MicroRNAs in skeletogenesis. Front Biosci. (Landmark Ed) *14*, 2757–2764.

20. Takamizawa, J., Konishi, H., Yanagisawa, K., Tomida, S., Osada, H., Endoh, H., Harano, T., Yatabe, Y., Nagino, M., Nimura, Y., et al. (2004). Reduced expression of the let-7 microRNAs in human lung cancers in association with shortened postoperative survival. Cancer Res. *64*, 3753–3756.

21. Wienholds, E., Kloosterman, W.P., Miska, E., Alvarez-Saavedra, E., Berezikov, E., de Bruijn, E., Horvitz, H.R., Kauppinen, S., and Plasterk, R.H.A. (2001). MicroRNA Expression in Zebrafish Embryonic Development. Science *309*, 310–311.

22. Miyaki, S., Sato, T., Inoue, A., Otsuki, S., Ito, Y., Yokoyama, S., Kato, Y., Takemoto, F., Nakasa, T., Yamashita, S., et al. (2010). MicroRNA-140 plays dual roles in both cartilage development and homeostasis. Genes Dev. *24*, 1173–1185.

23. Karlsen, T.A., Shahdadfar, A., and Brinchmann, J.E. (2011). Human primary articular chondrocytes, chondroblasts-like cells, and dedifferentiated chondrocytes: differences in gene, microRNA, and protein expression and phenotype. Tissue Eng. Part C Methods *17*, 219–227.

24. Yin, C.-M., Suen, W.-C.-W., Lin, S., Wu, X.-M., Li, G., and Pan, X.-H. (2017). Dysregulation of both miR-140-3p and miR-140-5p in synovial fluid correlate with osteoarthritis severity. Bone Joint Res. *6*, 612–618.

25. Ntoumou, E., Tzetis, M., Braoudaki, M., Lambrou, G., Poulou, M., Malizos, K., Stefanou, N., Anastasopoulou, L., and Tsezou, A. (2017). Serum microRNA array analysis identifies miR-140-3p, miR-33b-3p and miR-671-3p as potential osteoarthritis biomarkers involved in metabolic processes. Clin. Epigenetics *9*, 127.

26. Karlsen, T.A., Jakobsen, R.B., Mikkelsen, T.S., and Brinchmann, J.E. (2014). microRNA-140 targets RALA and regulates chondrogenic differentiation of human mesenchymal stem cells by translational enhancement of SOX9 and ACAN. Stem Cells Dev. *23*, 290–304.

27. Barter, M.J., Tselepi, M., Gómez, R., Woods, S., Hui, W., Smith, G.R., Shanley, D.P., Clark, I.M., and Young, D.A. (2015). Genome-Wide MicroRNA and Gene Analysis of Mesenchymal Stem Cell Chondrogenesis Identifies an Essential Role and Multiple Targets for miR-140-5p. Stem Cells *33*, 3266–3280.

28. Karlsen, T.A., de Souza, G.A., Ødegaard, B., Engebretsen, L., and Brinchmann, J.E. (2016). microRNA-140 Inhibits Inflammation and Stimulates Chondrogenesis in a Model of Interleukin 1β-induced Osteoarthritis. Mol. Ther. Nucleic Acids *5*, e373.

29. Wang, Z., Hu, J., Pan, Y., Shan, Y., Jiang, L., Qi, X., and Jia, L. (2018). miR-140-5p/miR-149 Affects Chondrocyte Proliferation, Apoptosis, and Autophagy by Targeting FUT1 in Osteoarthritis. Inflammation *41*, 959–971.

30. Zhai, H., Fesler, A., Ba, Y., Wu, S., and Ju, J. (2015). Inhibition of colorectal cancer stem cell survival and invasive potential by hsa-miR-140-5p mediated suppression of Smad2 and autophagy. Oncotarget *6*, 19735–19746.

31. Wei, R., Cao, G., Deng, Z., Su, J., and Cai, L. (2016). miR-140-5p attenuates chemotherapeutic drug-induced cell death by regulating autophagy through inositol 1,4,5-trisphosphate kinase 2 (IP3k2) in human osteosarcoma cells. Biosci. Rep. *36*, e00392.

32. Meng, Y., Gao, R., Ma, J., Zhao, J., Xu, E., Wang, C., and Zhou, X. (2017). MicroRNA-140-5p regulates osteosarcoma chemoresistance by targeting HMGN5 and autophagy OPEN. Sci. Rep. *7*, 416.

33. Jude, J.A., Dileepan, M., Subramanian, S., Solway, J., Panettieri, R.A., Jr., Walseth, T.F., and Kannan, M.S. (2012). miR-140-3p regulation of TNF-α-induced CD38 expression in human airway smooth muscle cells. Am. J. Physiol. Lung Cell. Mol. Physiol. *303*, L460–L468.

34. Takata, A., Otsuka, M., Kojima, K., Yoshikawa, T., Kishikawa, T., Yoshida, H., and Koike, K. (2011). MicroRNA-22 and microRNA-140 suppress NF-κB activity by regulating the expression of NF-κB coactivators. Biochem. Biophys. Res. Commun. *411*, 826–831.

35. Saba, R., Sorensen, D.L., and Booth, S.A. (2014). MicroRNA-146a: A Dominant, Negative Regulator of the Innate Immune Response. Front. Immunol. *5*, 578.

36. Taganov, K.D., Boldin, M.P., Chang, K.-J., and Baltimore, D. (2006). NF-kappaB-dependent induction of microRNA miR-146, an inhibitor targeted to signaling proteins of innate immune responses. Proc. Natl. Acad. Sci. USA *103*, 12481–12486.

37. Yamasaki, K., Nakasa, T., Miyaki, S., Ishikawa, M., Deie, M., Adachi, N., Yasunaga, Y., Asahara, H., and Ochi, M. (2009). Expression of MicroRNA-146a in osteoarthritis cartilage. Arthritis Rheum. *60*, 1035–1041.

38. Zou, W., Liu, T., Zhang, F., et al. (2015). An Investigation Focused on miR-146a and Autophagy in Chondrocytes under Hypoxia MicroRNA-146a Induced by Hypoxia Promotes Chondrocyte Autophagy through Bcl-2. Cell. Physiol. Biochem. *37*, 1442–1453.

39. Kristiansen, H., Gad, H.H., Eskildsen-Larsen, S., Despres, P., and Hartmann, R. (2011). The oligoadenylate synthetase family: an ancient protein family with multiple antiviral activities. J. Interferon Cytokine Res. *31*, 41–47.

40. Huber, M., Suprunenko, T., Ashhurst, T., Marbach, F., Raifer, H., Wolff, S., Strecker, T., Viengkhou, B., Jung, S.R., Obermann, H.L., et al. (2017). IRF9 Prevents CD8$^+$ T Cell Exhaustion in an Extrinsic Manner during Acute Lymphocytic Choriomeningitis Virus Infection. J. Virol. *91*, e01219–e17.

41. Virbasius, J.V., and Czech, M.P. (2016). Map4k4 Signaling Nodes in Metabolic and Cardiovascular Diseases. Trends Endocrinol. Metab. *27*, 484–492.

42. Murray, P.J. (2006). STAT3-mediated anti-inflammatory signalling. Biochem. Soc. Trans. *34*, 1028–1031.

43. Kondo, M., Yamaoka, K., Sakata, K., Sonomoto, K., Lin, L., Nakano, K., and Tanaka, Y. (2015). Contribution of the Interleukin-6/STAT-3 Signaling Pathway to Chondrogenic Differentiation of Human Mesenchymal Stem Cells. Arthritis Rheumatol. *67*, 1250–1260.

44. Hall, M.D., Murray, C.A., Valdez, M.J., and Perantoni, A.O. (2017). Mesoderm-specific Stat3 deletion affects expression of Sox9 yielding Sox9-dependent phenotypes. PLoS Genet. *13*, e1006610.

45. Greeve, I., Hermans-Borgmeyer, I., Brellinger, C., Kasper, D., Gomez-Isla, T., Behl, C., Levkau, B., and Nitsch, R.M. (2000). The human DIMINUTO/DWARF1

homolog seladin-1 confers resistance to Alzheimer's disease-associated neurodegeneration and oxidative stress. J. Neurosci. *20*, 7345–7352.

46. Bradley, E.W., and Drissi, M.H. (2010). WNT5A regulates chondrocyte differentiation through differential use of the CaN/NFAT and IKK/NF-kappaB pathways. Mol. Endocrinol. *24*, 1581–1593.

47. Saha, S., Harrison, S.H., and Chen, J.Y. (2009). Dissecting the human plasma proteome and inflammatory response biomarkers. Proteomics *9*, 470–484.

48. Endo, K., Takeshita, T., Kasai, H., Sasaki, Y., Tanaka, N., Asao, H., Kikuchi, K., Yamada, M., Chenb, M., O'Shea, J.J., and Sugamura, K. (2000). STAM2, a new member of the STAM family, binding to the Janus kinases. FEBS Lett. *477*, 55–61.

49. Yoon, B.S., Pogue, R., Ovchinnikov, D.A., Yoshii, I., Mishina, Y., Behringer, R.R., and Lyons, K.M. (2006). BMPs regulate multiple aspects of growth-plate chondrogenesis through opposing actions on FGF pathways. Development *133*, 4667–4678.

50. Parafioriti, A., del Bianco, S., Barisani, D., Armiraglio, E., Peretti, G., and Albisetti, W. (2009). Increased p21 expression in chondrocytes of achondroplasic children independently from the presence of the G380R FGFR3 mutation. J. Orthop. Sci. *14*, 623–630.

51. Legeai-Mallet, L., Benoist-Lasselin, C., Munnich, A., and Bonaventure, J. (2004). Overexpression of FGFR3, Stat1, Stat5 and p21Cip1 correlates with phenotypic severity and defective chondrocyte differentiation in FGFR3-related chondrodysplasias. Bone *34*, 26–36.

52. Liao, W., Li, Z., Li, T., Zhang, Q., Zhang, H., and Wang, X. (2018). Proteomic analysis of synovial fluid in osteoarthritis using SWATH-mass spectrometry. Mol. Med. Rep. *17*, 2827–2836.

53. Sieker, J.T., Proffen, B.L., Waller, K.A., Chin, K.E., Karamchedu, N.P., Akelman, M.R., Perrone, G.S., Kiapour, A.M., Konrad, J., Murray, M.M., and Fleming, B.C. (2018). Transcriptional profiling of articular cartilage in a porcine model of early post-traumatic osteoarthritis. J. Orthop. Res. *36*, 318–329.

54. Norrie, J.L., Li, Q., Co, S., Huang, B.L., Ding, D., Uy, J.C., Ji, Z., Mackem, S., Bedford, M.T., Galli, A., et al. (2016). PRMT5 is essential for the maintenance of chondrogenic progenitor cells in the limb bud. Development *143*, 4608–4619.

55. Orlando, U.D., Castillo, A.F., Dattilo, M.A., Solano, A.R., Maloberti, P.M., and Podesta, E.J. (2015). Acyl-CoA synthetase-4, a new regulator of mTOR and a potential therapeutic target for enhanced estrogen receptor function in receptor-positive and -negative breast cancer. Oncotarget *6*, 42632–42650.

56. Needham, B.E., Wlodek, M.E., Ciccotosto, G.D., Fam, B.C., Masters, C.L., Proietto, J., Andrikopoulos, S., and Cappai, R. (2008). Identification of the Alzheimer's disease amyloid precursor protein (APP) and its homologue APLP2 as essential modulators of glucose and insulin homeostasis and growth. J. Pathol. *215*, 155–163.

57. Maliekal, P., Sokolova, T., Vertommen, D., Veiga-da-Cunha, M., and Van Schaftingen, E. (2007). Molecular identification of mammalian phosphopentomutase and glucose-1,6-bisphosphate synthase, two members of the alpha-D-phosphohexomutase family. J. Biol. Chem. *282*, 31844–31851.

58. Quintana, A.M., Geiger, E.A., Achilly, N., Rosenblatt, D.S., Maclean, K.N., Stabler, S.P., Artinger, K.B., Appel, B., and Shaikh, T.H. (2014). Hcfc1b, a zebrafish ortholog of HCFC1, regulates craniofacial development by modulating mmachc expression. Dev. Biol. *396*, 94–106.

59. Gori, F., Zhu, E.D., and Demay, M.B. (2009). Perichondrial expression of Wdr5 regulates chondrocyte proliferation and differentiation. Dev. Biol. *329*, 36–43.

60. Notari, M., Hu, Y., Koch, S., Lu, M., Ratnayaka, I., Zhong, S., Baer, C., Pagotto, A., Goldin, R., Salter, V., et al. (2011). Inhibitor of apoptosis-stimulating protein of p53 (iASPP) prevents senescence and is required for epithelial stratification. Proc. Natl. Acad. Sci. USA *108*, 16645–16650.

61. Lankat-Buttgereit, B., and Göke, R. (2003). Programmed cell death protein 4 (pdcd4): a novel target for antineoplastic therapy? Biol. Cell *95*, 515–519.

62. Steiglitz, B.M., Kreider, J.M., Frankenburg, E.P., Pappano, W.N., Hoffman, G.G., Meganck, J.A., Liang, X., Höök, M., Birk, D.E., Goldstein, S.A., and Greenspan, D.S. (2006). Procollagen C proteinase enhancer 1 genes are important determinants of the mechanical properties and geometry of bone and the ultrastructure of connective tissues. Mol. Cell. Biol. *26*, 238–249.

63. Zhao, C., Li, T., Han, B., Yue, W., Shi, L., Wang, H., Guo, Y., and Lu, Z. (2016). DDAH1 deficiency promotes intracellular oxidative stress and cell apoptosis via a miR-21-dependent pathway in mouse embryonic fibroblasts. Free Radic. Biol. Med. *92*, 50–60.

64. Ruefli-Brasse, A.A., Lee, W.P., Hurst, S., and Dixit, V.M. (2004). Rip2 participates in Bcl10 signaling and T-cell receptor-mediated NF-kappaB activation. J. Biol. Chem. *279*, 1570–1574.

65. Bahram, S., Arnold, D., Bresnahan, M., Strominger, J.L., and Spies, T. (1991). Two putative subunits of a peptide pump encoded in the human major histocompatibility complex class II region. Proc. Natl. Acad. Sci. USA. *88*, 10094–10098.

66. Sui, L., Zhang, W., Liu, Q., Chen, T., Li, N., Wan, T., Yu, M., and Cao, X. (2003). Cloning and functional characterization of human septin 10, a novel member of septin family cloned from dendritic cells. Biochem. Biophys. Res. Commun. *304*, 393–398.

67. Hambleton, S., Goodbourn, S., Young, D.F., Dickinson, P., Mohamad, S.M., Valappil, M., McGovern, N., Cant, A.J., Hackett, S.J., Ghazal, P., et al. (2013). STAT2 deficiency and susceptibility to viral illness in humans. Proc. Natl. Acad. Sci. USA *110*, 3053–3058.

68. Porté, S., Valencia, E., Yakovtseva, E.A., Borràs, E., Shafqat, N., Debreczeny, J.E., Pike, A.C., Oppermann, U., Farrés, J., Fita, I., and Parés, X. (2009). Three-dimensional structure and enzymatic function of proapoptotic human p53-inducible quinone oxidoreductase PIG3. J. Biol. Chem. *284*, 17194–17205.

69. Wang, D. (2008). Discrepancy between mRNA and protein abundance: insight from information retrieval process in computers. Comput. Biol. Chem. *32*, 462–468.

70. Loeser, R.F., Collins, J.A., and Diekman, B.O. (2016). Ageing and the pathogenesis of osteoarthritis. Nat. Rev. Rheumatol. *12*, 412–420.

71. Mobasheri, A., Rayman, M.P., Gualillo, O., Sellam, J., van der Kraan, P., and Fearon, U. (2017). The role of metabolism in the pathogenesis of osteoarthritis. Nat. Rev. Rheumatol. *13*, 302–311.

72. Qian, M., Fang, X., and Wang, X. (2017). Autophagy and inflammation. Clin. Transl. Med. *6*, 24.

73. Harris, J., Hartman, M., Roche, C., Zeng, S.G., O'Shea, A., Sharp, F.A., Lambe, E.M., Creagh, E.M., Golenbock, D.T., Tschopp, J., et al. (2011). Autophagy controls IL-1beta secretion by targeting pro-IL-1beta for degradation. J. Biol. Chem. *286*, 9587–9597.

74. Goldring, M.B., and Otero, M. (2011). Inflammation in osteoarthritis. Curr. Opin. Rheumatol. *23*, 471–478.

75. Harris, J. (2013). Autophagy and IL-1 Family Cytokines. Front. Immunol. *4*, 83.

76. Reed, K.N., Wilson, G., Pearsall, A., and Grishko, V.I. (2014). The role of mitochondrial reactive oxygen species in cartilage matrix destruction. Mol. Cell. Biochem. *397*, 195–201.

77. Kim, J., Xu, M., Xo, R., Mates, A., Wilson, G.L., Pearsall, A.W., 4th, and Grishko, V. (2010). Mitochondrial DNA damage is involved in apoptosis caused by pro-inflammatory cytokines in human OA chondrocytes. Osteoarthritis Cartilage *18*, 424–432.

78. Vidigal, J.A., and Ventura, A. (2015). The biological functions of miRNAs: lessons from in vivo studies. Trends Cell Biol. *25*, 137–147.

79. de Pontual, L., Yao, E., Callier, P., Faivre, L., Drouin, V., Cariou, S., Van Haeringen, A., Geneviève, D., Goldenberg, A., Oufadem, M., et al. (2011). Germline deletion of the miR-17∼92 cluster causes skeletal and growth defects in humans. Nat. Genet. *43*, 1026–1030.

80. Tan, C.L., Plotkin, J.L., Venø, M.T., von Schimmelmann, M., Feinberg, P., Mann, S., Handler, A., Kjems, J., Surmeier, D.J., O'Carroll, D., et al. (2013). MicroRNA-128 governs neuronal excitability and motor behavior in mice. Science *342*, 1254–1258.

81. Wang, D., Zhang, Z., O'Loughlin, E., Wang, L., Fan, X., Lai, E.C., and Yi, R. (2013). MicroRNA-205 controls neonatal expansion of skin stem cells by modulating the PI(3)K pathway. Nat. Cell Biol. *15*, 1153–1163.

82. Kobayashi, H., Hirata, M., Saito, T., Itoh, S., Chung, U.I., and Kawaguchi, H. (2013). Transcriptional induction of ADAMTS5 protein by nuclear factor-κB (NF-κB) family member RelA/p65 in chondrocytes during osteoarthritis development. J. Biol. Chem. *288*, 28620–28629.

83. Nakagawa, K., Sakiyama, H., Tsuchida, T., Yamaguchi, K., Toyoguchi, T., Masuda, R., and Moriya, H. (1999). Complement C1s activation in degenerating articular cartilage of rheumatoid arthritis patients: immunohistochemical studies with an active form specific antibody. Ann. Rheum. Dis. *58*, 175–181.

84. Rockel, J.S., Bernier, S.M., and Leask, A. (2009). Egr-1 inhibits the expression of extracellular matrix genes in chondrocytes by TNFalpha-induced MEK/ERK signalling. Arthritis Res. Ther. *11*, R8.

85. Qian, Y., Wang, G., Xue, F., Chen, L., Wang, Y., Tang, L., and Yang, H. (2017). Genetic association between TAP1 and TAP2 polymorphisms and ankylosing spondylitis: a systematic review and meta-analysis. Inflamm. Res. *66*, 653–661.

86. Mostowy, S., Bonazzi, M., Hamon, M.A., Tham, T.N., Mallet, A., Lelek, M., Gouin, E., Demangel, C., Brosch, R., Zimmer, C., et al. (2010). Entrapment of intracytosolic bacteria by septin cage-like structures. Cell Host Microbe *8*, 433–444.

87. Millward-Sadler, S.J., Khan, N.S., Bracher, M.G., Wright, M.O., and Salter, D.M. (2006). Roles for the interleukin-4 receptor and associated JAK/STAT proteins in human articular chondrocyte mechanotransduction. Osteoarthritis Cartilage *14*, 991–1001.

88. Richard, M., Drouin, R., and Beaulieu, A.D. (1998). ABC50, a novel human ATP-binding cassette protein found in tumor necrosis factor-α-stimulated synoviocytes. Genomics *53*, 137–145.

89. Powell, T.R., Tansey, K.E., Breen, G., Farmer, A.E., Craig, I.W., Uher, R., McGuffin, P., D'Souza, U.M., and Schalkwyk, L.C. (2013). ATP-binding cassette sub-family F member 1 (ABCF1) is identified as a putative therapeutic target of escitalopram in the inflammatory cytokine pathway. J. Psychopharmacol. (Oxford) *27*, 609–615.

90. Wilcox, S.M. (2010). The function of ABCF1 in immunity and mouse development. PhD Thesis (University of British Columbia).

91. Jun, J.C., Cominelli, F., and Abbott, D.W. (2013). RIP2 activity in inflammatory disease and implications for novel therapeutics. J. Leukoc. Biol. *94*, 927–932.

92. Jurynec, M.J., Sawitzke, A.D., Beals, T.C., Redd, M.J., Stevens, J., Otterud, B., Leppert, M.F., and Grunwald, D.J. (2018). A hyperactivating proinflammatory RIPK2 allele associated with early-onset osteoarthritis. Hum. Mol. Genet. *27*, 2383–2391.

93. Andreas, K., Lübke, C., Häupl, T., Dehne, T., Morawietz, L., Ringe, J., Kaps, C., and Sittinger, M. (2008). Key regulatory molecules of cartilage destruction in rheumatoid arthritis: an in vitro study. Arthritis Res. Ther. *10*, R9.

94. Xu, J., Jiang, C., Zhu, W., Wang, B., Yan, J., Min, Z., Geng, M., Han, Y., Ning, Q., Zhang, F., et al. (2015). NOD2 pathway via RIPK2 and TBK1 is involved in the aberrant catabolism induced by T-2 toxin in chondrocytes. Osteoarthritis Cartilage *23*, 1575–1585.

95. Zhang, X., Wang, C., Zhao, J., Xu, J., Geng, Y., Dai, L., Huang, Y., Fu, S.C., Dai, K., and Zhang, X. (2017). miR-146a facilitates osteoarthritis by regulating cartilage homeostasis via targeting Camk2d and Ppp3r2. Cell Death Dis. *8*, e2734.

96. Loeser, R.F. (2011). Aging and osteoarthritis. Curr. Opin. Rheumatol. *23*, 492–496.

97. Rubinsztein, D.C., Mariño, G., and Kroemer, G. (2011). Autophagy and aging. Cell *146*, 682–695.

98. Caramés, B., Olmer, M., Kiosses, W.B., and Lotz, M.K. (2015). The relationship of autophagy defects to cartilage damage during joint aging in a mouse model. Arthritis Rheumatol. *67*, 1568–1576.

99. Zhang, Y., Vasheghani, F., Li, Y.H., Blati, M., Simeone, K., Fahmi, H., Lussier, B., Roughley, P., Lagares, D., Pelletier, J.P., et al. (2015). Cartilage-specific deletion of mTOR upregulates autophagy and protects mice from osteoarthritis. Ann. Rheum. Dis. *74*, 1432–1440.

100. Ramakrishnan, P.S., Brouillette, M.J., and Martin, J.A. (2013). Oxidative Conditioning and Treatment for Osteoarthritis. In Studies on Arthritis and Joint Disorders, M. Alcaraz, O. Gualillo, and O. Sánchez-Pernaute, eds. (Springer New York), pp. 311–332.

101. Sasaki, H., Takayama, K., Matsushita, T., Ishida, K., Kubo, S., Matsumoto, T., Fujita, N., Oka, S., Kurosaka, M., and Kuroda, R. (2012). Autophagy modulates osteoarthritis-related gene expression in human chondrocytes. Arthritis Rheum. *64*, 1920–1928.

102. Andersson, H.C., Kratz, L., and Kelley, R. (2002). Desmosterolosis presenting with multiple congenital anomalies and profound developmental delay. Am. J. Med. Genet. *113*, 315–319.

103. Mirza, R., Qiao, S., Tateyama, K., Miyamoto, T., Xiuli, L., and Seo, H. (2012). 3β-Hydroxysterol-Delta24 reductase plays an important role in long bone growth by protecting chondrocytes from reactive oxygen species. J. Bone Miner. Metab. *30*, 144–153.

104. Shi, L., Zhao, C., Wang, H., Lei, T., Liu, S., Cao, J., and Lu, Z. (2017). Dimethylarginine Dimethylaminohydrolase 1 Deficiency Induces the Epithelial to Mesenchymal Transition in Renal Proximal Tubular Epithelial Cells and Exacerbates Kidney Damage in Aged and Diabetic Mice. Antioxid. Redox. Signal *27*, 1347–1360.

105. Schallmoser, K., and Strunk, D. (2009). Preparation of pooled human platelet lysate (pHPL) as an efficient supplement for animal serum-free human stem cell cultures. J. Vis. Exp. *32*, e1523.

OMTN, Volume *17*

Supplemental Information

Multi-pathway Protective Effects of MicroRNAs on Human Chondrocytes in an *In Vitro* Model of Osteoarthritis

Rua Nader Al-Modawi, Jan E. Brinchmann, and Tommy A. Karlsen

Supplementary 1: Shared proteins between the three miRNAs

Common downregulated between miR-140-5p and miR140-3p	Common upregulated between miR-140-5p and miR140-3p	Common downregulated between miR-140-5p and miR146a	Common upregulated between miR-140-3p and miR146a
STA5A	STAT3	HCFC1	PCOC1
C1R	MEP50		
	NUP93		

Supplementary 2: Taqman probes, miRNA mimics and antibodies

Taqman Assays

Official symbol	Official full name	Company	Cat no.
IL6	Interleukin-6	Thermo Fisher Scientific	Hs00985639_m1
IL8	Interleukin-8	Thermo Fisher Scientific	Hs00174103_m1
IL1β	Interleukin 1-beta	Thermo Fisher Scientific	Hs01555410_m1
MMP13	Matrix metallopeptidase 13	Thermo Fisher	Hs00233992_m1
ADAMTS5	A disintigrin and metallopeptidase with thrombospondin type 1 motif, 5	Thermo Fisher Scientific	Hs00199841_m1
GAPDH	Glyceraldehyde-3-phosphate dehydrogenase	Thermo Fisher Scientific	Hs99999905_m1
miR-140-5p	microRNA 140-5p	Thermo Fisher Scientific	001187
U18	U18 small nuclear RNA	Thermo Fisher Scientific	001204
miR140-3p	microRNA 140-3p	Thermo Fisher Scientific	002234
miR146a	microRNA 146a	Thermo Fisher Scientific	00468
GABARAP	Gamma-aminobutyric acid receptor-associated protein	Thermo Fisher Scientific	Hs01572686_m1
CIR	Complement C1r subcomponent	Thermo Fisher Scientific	Hs00354278_m1
STAT5	Signal transducer and activator of transcription 5A	Thermo Fisher Scientific	Hs00559637_g1
RALA	Ras-related protein Ral-A	Thermo Fisher	Hs00800233_s1

		Scientific	
DHCR24	Delta(24)-sterol reductase	Thermo Fisher Scientific	Hs00207388_m1
LAMTOR5	Ragulator complex protein LAMTOR5	Thermo Fisher Scientific	Hs00246261_m1
BROX	BRO1 domain-containing protein	Thermo Fisher Scientific	Hs01013625_m1
DDAH1	N(G),N(G)-dimethylarginine dimethylaminohydrolase 1	Thermo Fisher Scientific	Hs00201707_m1
RIPK2	Receptor-interacting serine/threonine-protein kinase 2	Thermo Fisher Scientific	Hs01572686_m1
STAT2	Signal transducer and activator of transcription 2	Thermo Fisher Scientific	Hs01013115_g1
TP5313	Quinone oxidoreductase PIG3	Thermo Fisher Scientific	Hs00936519_m1

<u>microRNA mimics</u>

**Name**		**Company**	**Cat no.**
Pre-negative control no. 1		Thermo Fisher Scientific	AM17110
Pre-miR-140-5p	microRNA-140-5p	Thermo Fisher Scientific	PM10205
Pre-miR-140-3p	microRNA-140-3p	Thermo Fisher Scientific	PM12503
Pre-miR-146a	microRNA-146a	Thermo Fisher Scientific	PM10722

<u>Antibodies (western blot)</u>

**Primary antibodies**	**Concentration**	**Company**	**Cat.no**
Mouse anti-ACTB	1:1000	Abcam	ab8226
Rabbit anti-IL8	1:1000	Millipore	AB1427
Rabbit anti-IL6	1:1000	Abcam	Ab32530
Mouse anti-TRAF6	1:1000	Abcam	Ab40675
Rabbit anti-RALA	1:2000	Abcam	Ab126627
Rabbit anti-GBRAP	1:1000	Cell signaling	# 13733
Rabbit anti-RIPK2	1:1000	Abcam	Ab8428

Rabbit anti-NFκB-P65	1:1600	Abcam	Ab16502
Rabbit anti-LAMTORC	1:1000	Cell signaling	#14633
Rabbit anti-PDCD4	1:10000	Abcam	Ab80590
**<u>Secondary antibodies</u>**			
Goat anti-Rabbit (HRP)	1:5000<br>1:2000(when used with primary rabbit anti-PDCD4)	Vector labs	PI-1000
Horse anti-Mouse (HRP)	1:2000	Vector labs	PI-2000
